# The Usefulness of Intraoperative PTH as a Predictor for Successful Parathyroidectomy in Secondary Hyperparathyroidism

**DOI:** 10.3389/fsurg.2021.696469

**Published:** 2021-06-28

**Authors:** Karla Verónica Chávez, Horacio Márquez-González, Mariana Chavez-Tostado

**Affiliations:** ^1^Department of Human Reproduction, Health Sciences University Center, University of Guadalajara, Guadalajara, Mexico; ^2^Hospital de Cardiología, Centro Médico Nacional “Siglo XXI”, México City, Mexico

**Keywords:** secondary hyperparathyroidism, intraoperative paratohormone, parathyroidectomy, renal hyperparathyroidism, paratohormone

## Abstract

**Introduction:** Secondary hyperparathyroidism (SHPT) is a multisystemic syndrome that affects calcium and bone homeostasis in patients with chronic kidney disease (CKD). Despite medical treatment, 1–2% of patients require parathyroidectomy annually. The use of an intraoperative parathormone protocol (IOPTH) to predict cure is still in debate, due to the lack of standardized protocols, the use of different assays, and uneven PTH clearance. This study aimed to determine the diagnostic accuracy of an IOPTH in patients with SHPT for predicting successful surgery after parathyroidectomy.

**Methods:** About 30 patients were enrolled. A prospective observational study (cohort) was performed in patients who were submitted to parathyroidectomy by an endocrine surgeon for SHPT. All were submitted to a bilateral neck exploration with a subtotal parathyroidectomy. Three IOPTH determinations were withdrawn: at anesthetic induction (PTH_0_), 15 min (PTH_15_), and 30 min (PTH_30_) after completion of gland resection. Another sample was taken 24 h after the procedure (PTH_24_), values <150 pg/mL defined a successful surgery, and patients were assigned to the success or failure group. IOPTH drop was analyzed to predict successful surgery with drops of 70 and 90% at 15 and 30 min, respectively.

**Results:** A total of 26 patients were included, 19 patients were in the successful group. IOPTH showed a significant difference between groups in their absolute PTH_15_ and PTH_30_ values. A significant difference was also found in their PTH drop at 30 min (81 vs. 91%, *p* = 0.08). For predicting a successful surgery, having a PTH drop >90% at 30 min was the most significant factor [Odds Ratio (OR) 3.0 (1.5–4) IC 95%].

**Conclusions:** This study points toward a stricter and staggered IOPTH protocol to predict a successful surgery. Our results suggest taking a PTH_15_ expecting a PTH drop of >90%. If this is not achieved, reexploration and a PTH_30_ sample are suggested to accurately predict success.

## Introduction

Renal or secondary hyperparathyroidism (SHPT) is a multisystemic syndrome in which calcium and bone homeostasis are affected as a result of chronic 25-OH-Vitamin D deficiency and hyperphosphatemia in patients with chronic kidney disease (CKD) (1). This imbalance is present in almost every patient with CKD despite the medical and technological improvements for their treatment (2).

About 4–14% of all patients with SHPT do not respond successfully to optimal medical treatment (3) and 1–2% require parathyroidectomy each year (1). Nowadays, parathyroidectomy is a valid, remedial procedure for the treatment of SHPT; however, timing, extent, type of surgery, and the implementation of an intraoperative parathormone protocol (IOPTH) still presents a common topic for debate between endocrine surgeons (4).

Intraoperative parathormone protocol has proven its usefulness as an adjunct during surgery for primary hyperparathyroidism (5). However, the value of IOPTH in parathyroid surgery for SHPT has not been established, due to the lack of standardized protocols, use of different assays with different specificity, and the uneven reduction kinetics in patients with impaired renal function (6).

This study aimed to determine the diagnostic accuracy of an IOPTH protocol in patients with SHPT for predicting successful surgery to avoid recurrence after parathyroidectomy.

## Materials and Methods

A total of 30 patients were included, between July 2017 and November 2019 in a second-level Hospital. A prospective observational study (cohort) was performed in patients who were summited to surgery by an endocrine surgeon for management of uncontrolled SHPT, referred by nephrology after unsuccessful medical therapy. Preoperative evaluation for surgery included complete laboratory testing and cardiac risk assessment. All patients signed informed consent; the study was approved by the Education and Investigation department.

All were summited to a bilateral neck exploration with a subtotal parathyroidectomy, leaving approximately 50 mg of parathyroid tissue *in situ*. Cervical thymectomy was performed only in cases with a missing gland. After surgery, analgesics were given for 5 days, and patients were discharged when calcium was above 8.0 mg/dL.

Exposure variables were determined with three IOPTH determinations that were withdrawn through a peripheric venous line in the forearm of the patient at different times: at anesthetic induction (PTH_0_), 15 min (PTH_15_), and 30 min (PTH_30_) after resection of parathyroid glands. Another sample was taken 24 h after the procedure (PTH_24_), values <150 pg/mL defined a successful outcome, and patients were assigned to the success or failure group.

Intraoperative parathormone protocol drop between PTH_0_ and subsequent measures was nominated PTH drop (ΔPTH). ΔPTH was analyzed to predict success with drops of 70 and 90% at 15 and 30 min, respectively. PTH levels were measured using Beckman Coulter® Access Immunoassay systems® (Brea, California, US).

Inclusion criteria included CKD with PTH >800 pg/mL and SHPT symptoms or asymptomatic patients with PTH >1,000 pg/mL who were resistant to treatment, age between 18 and 90 years old, compliance to sign the informed consent. Exclusion criteria were patients who respond adequately to medical treatment, those with incomplete IOPTH protocol, and those who did not meet any other inclusion criteria.

### Statistical Analysis

The qualitative variables were expressed in absolute frequencies and percentages, quantitative variables in median and interquartile ranges. Bivariate analysis was performed between subjects with and without response to treatment. The risk was calculated with OR, and the best cut-off point was calculated to predict therapeutic failure with the ROC curve and PTH measurements (0, 15, and 30 min). The study involved <30 subjects, the Mantel and Haenszal X tests were used to identify confounding variables, and adjusting the ORs were adjusted by a confounder. A *p-*value of <0.05 was considered statistically significant.

The statistical program used was SPSS for MAC version 22.

## Results

A total of 30 patients were enrolled, four had to be excluded because of incomplete IOPTH protocol. Nineteen of the 26 patients were men (73%) and seven women (27%), with a median age of 37 years (IQR 29–51yr).

Renal substitution therapy was given to 22 patients (85%), 15 of them with peritoneal dialysis (68.1%) and seven with hemodialysis (31.8%). We only had three post-transplant patients, one of them with a failed graft. Mean preoperative PTH was 1633.9 pg/mL, the complete preoperative workup resumes in Table 1.

**Table 1:** Preoperative biochemical workup.

	**Mean**	**Range**	**SD±**
PTH (pg/mL)	1633.9	908–3,400	666
Calcium (mg/dL)	9.36	7.10–12.28	1.08
Phosphate (mg/dL)	6.01	2.20–9.60	1.89
Albumin (mg/dL)	3.45	2.5–4.4	0.50
Magnesium (mg/dL)	2.14	1.4–3.19	0.42
Creatinine (mg/dL)	11.65	1.2–22	5.51
Alkaline phosphatase (UI/L)	383.17	57–2,154	569.82

*SD, standard deviation*.

At 24 h 19 patients had PTH_24_ <150 pg/mL (73%) and were considered the successful group, whereas the other six patients fail to comply with this and were assigned to the failure group. There were no differences between the successful and failure groups in terms of demographic or preoperative variables, nor the postoperative complications (Tables 2, 3).

**Table 2:** Demographic variables in both groups.

	**Failure**		**Successful**		***p***
	***n***	**%**	***n***	**%**	
**Sex**					
Female	3	43	4	21	0.2
Male	4	57	15	79	
Total	7	26	19	73	
**KDOQI stage**					
III	0	0	1	5	0.6
IV	0	0	1	5	
V	7	100	17	89	
**Renal replacement therapy**					
Hemodyalisis	2	29	5	33	0.8
Peritoneal	5	71	10	67	
**Outcomes**					
Hungry bone syndrome	1	14	2	11	0.7
Death	1	14	2	11	0.7
Recurrence	1	14	0	0	0.09

**Table 3:** Biochemical preoperative values in both groups.

	**Failure**			**Successful**			
	**Median**	**p25**	**p75**	**Median**	**p25**	**p75**	***p***
**Preoperative values**							
Age (years)	31	28	44	40	29	52	0.4
PTH (pg/mL)	1,857	1,062	2,491	1,414	1,094	1,572	0.3
Ca (mg/dL)	9.2	8.7	9.8	8.65	7.85	10	0.04^*^
PO4 (mg/dL)	5.8	5.3	8	5.8	5.05	7	0.4
ALB (mg/dL)	3.3	3	3.7	3.45	3.1	3.95	0.5
MG (mg/dL)	2.1	1.94	2.41	1.97	1.87	2.49	1

* *Statistically significant*.

Intraoperative parathormone protocol showed a significant difference between groups in their absolute PTH_15_ and PTH_30_ values. A significant difference was also found in ΔPTH_30_ (81 vs. 91%, *p* = 0.08) (Table 4).

**Table 4:** Comparison of Intraoperative parathormone (PTH) between groups.

	**Failure**			**Successful**			
	**Median**	**p25**	**p75**	**Median**	**p25**	**p75**	***p***
**Intraoperative variables**							
PTH_0_	1,643	1,002	2,051	1,137	738	2,072	0.3
PTH_15_	320	225	479	146	107	252	0.025^*^
PTH_30_	311	210	393	121	76	179	0.007^*^
PTH drop (15–30) %	0.07	0.03	0.18	0.18	0.09	0.35	0.1
PTH drop (0–30 min) %	0.81	0.78	0.9	0.91	0.86	0.94	**0.08**
PTH24h	207	166	263	46	10	66	<0.0001^*^

* *Statistically significant*.

*Significant values are in bold*.

**Figure 1:**
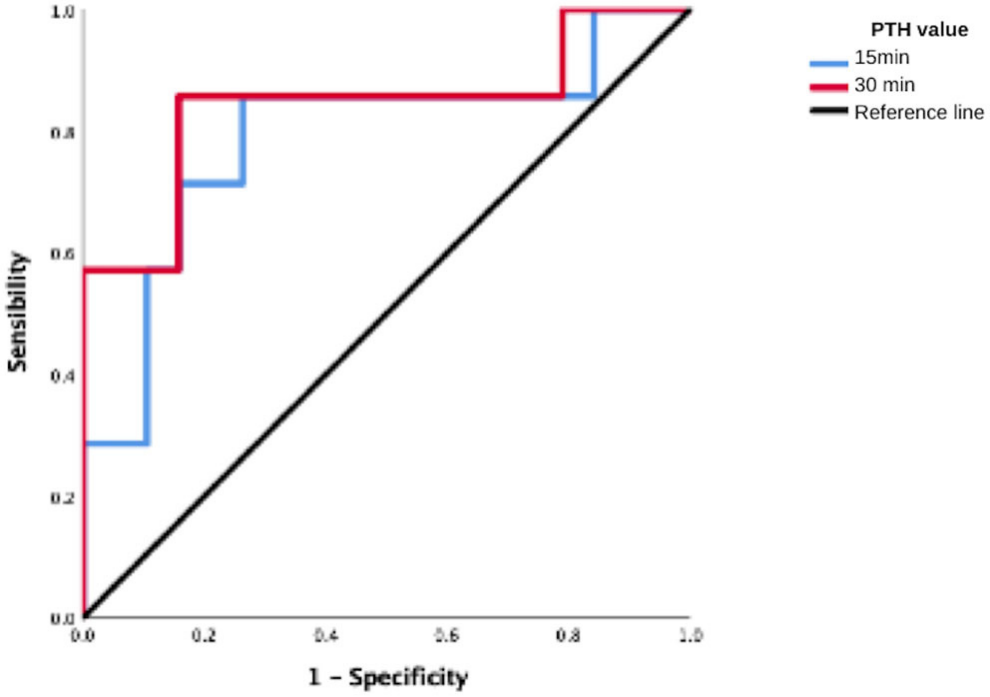
ROC curve to predict 24 h successful surgery with a >90% PTH drop.

When analyzing the risk for successful surgery, a ΔPTH >70% was not significant neither at 15 or 30 min [OR 1.2 (0.9–2.5) IC 95%]. But when having a ΔPTH >90% at 30 min, the OR was statistically significant [OR 3.0 (1.5–4) IC 95%] as described in Table 5 and Figure 1. As well, patients in the successful group had a ΔPTH >90% at 30 min in 85% cases, compared with only 73% in the failure group.

**Table 5:** Area under the curve (AUC) for predicting successful surgery.

	**Groups**		
	**Succesful *n* (%)**	**Failure*n* (%)**	**OR (IC-95%)**
ΔPTH _15_ > 70%	18 (94.7)	6 (85.7)	1.4 (0.7–9)
ΔPTH _30_ > 70%	18 (94.7)	6 (85.7)	1.2 (0.9–2.5)
ΔPTH _15_ > 90%	6 (31.6)	0	1.2 (0.9–1.6)
ΔPTH _30_ > 90%	10 (52.6)	1 (14.3)	**3 (1.5**–**4)**

*OR bivariate analysis*.

*Significant values are in bold*.

Intraoperative findings included an incidental synchronic parathyroid adenoma in one patient and another had an intra thyroidal parathyroid. At least four glands were identified in all patients. After surgery, 96% of patients received intravenous calcium gluconate infusion. The most common postoperative complication was severe hypocalcemia which was present in five patients (16.6%), three patients had hungry bone syndrome (10%). One patient had to be reoperated for postoperative bleeding. Mean hospital stay was 5.3 days (2–22 days), at discharge patients were taking a mean of 5.8 gr of calcium carbonate (0–15 gr) and 690 mcg of calcitriol (250–1,500 mcg). Three patients died during follow-up, non-surgery related. Persistent SHPT presented in one patient in the failure group during follow-up, her ΔPTH_30_ had an 89.7% drop and her PTH_24_ was 166 pg/mL.

## Discussion

Different authors have tried to establish a valid IOPTH protocol in patients with CKD. In our study, we performed an IOPTH protocol with two post-resection values and a confirmatory sample, we used these measures to validate which IOPTH protocol is better to predict a successful surgery without recurrence.

Hiramitsu et al. (7) retrospectively predetermined a cut-off for IOPTH drop of 70% 10 min after baseline and a value <60 pg/m PTH on a postoperative day 1 as an indicator for a successful outcome in 226 patients. Those with >60 pg/m 24 h after surgery had a significantly higher reoperation rate (13.0 vs. 0.5%, *p* = 0.003). Sensitivity, specificity, and accuracy of >70% IOPTH drop were 97.5, 52.2, and 92.9%, respectively. In another study by Seehofer et al. (8), considered two criteria for IOPTH in 153 patients: a drop <150 pg/mL 15 min after the resection and a drop >70% from preoperative baseline. If both were present an operative success was predicted in 98.7%.

In both of these studies (7, 8), a PTH drop of 70% was used to predict a successful surgery, we found a very low predictive value with this threshold as seen in Table 5. Stricter protocols have been described, Vulpio et al. (9) used a PTH drop >80% from a basal sample at 20 or 30 min, concluding that the combined PTH drop <88% at 30 min after excision or PTH ≥166 pg/mL is extremely accurate to predict the persistence of SHPT. Accordingly, in our study most patients with a ΔPTH >90% at 30 min had successful surgery (85%) and none of them presented recurrence.

Most previous reports use intraoperative samples at 10 and 15 min post-resection (10). We performed an extended protocol with two IOPTH samples 15 and 30 min after resection, to capture patients with a more delayed PTH clearance. To confirm this, we observed that the PTH_30_ had a better predictive value for a successful surgery compared to the PTH_15_ sample. Similarly, Lokey et al. (10) took IOPTH samples at induction, 20 and 40 min post-resection aiming a drop of 50%, they obtain a positive predictive value of 93% and a sensitivity of 96% for the cure at 20 min.

In this matter, it is important to consider that performing a PTH_30_ or a more extended protocol and then wait for the results takes valuable time during which the patient is under general anesthesia with extended OR time. To reduce the need of investing this much time, an interesting protocol was described by Barczyński et al. (11) who took a sample at 10 min looking for a drop of >60%, otherwise, a 20 min sample was taken for a further >80% drop, if this was not reached re-exploration was performed; this procedure had an impact in the surgery decision making of 15.7% of patients and an accuracy of 96%. In the same way, we did not find a significant difference in ΔPTH between the 15 and 30 min sample, therefore, we agree that a sample taken 15 min post-resection can be used intraoperatively as a sensitive tool for reexploring the neck when having <90% drop, whereas the PTH_30_ may be used to confirm a successful surgery.

## Conclusions

This study points toward a stricter and staggered IOPTH protocol, to achieve valid information to predict a successful surgery, hence, less recurrence and better quality of life. Our results suggest taking a PTH_15_ expecting a PTH drop >90%, if not achieved, reexploring and PTH_30_ sample is suggested to predict success accurately. However, we consider that the sample size may be a sample bias, therefore, we expect soon enough to validate strong evidence of using an IOPTH protocol in these patients as for primary hyperparathyroidism, for these matters, more studies and uniformity is needed in future investigations.

We must never lose out of sight that the only cure for these patients is normalizing renal function, as this disease is truly secondary for their CKD and until this is not treated, they will continue with hormonal hyperfunction, the mineral bone disease will persist, and other complications will eventually present.

## Data Availability Statement

The original contributions generated for this study are included in the article/supplementary materials, further inquiries can be directed to the corresponding author/s.

## Ethics Statement

The studies involving human participants were reviewed and approved by Hospital General Tlahuac Ethics Comitee. The patients/participants provided their written informed consent to participate in this study.

## Author Contributions

KC and HM-G: conception and design. KC: administrative support and provision of study materials or patients. KC and MC-T: collection and assembly of data. HM-G and MC-T: data analysis and interpretation. All authors: manuscript writing and final approval of manuscript.

## Conflict of Interest

The authors declare that the research was conducted in the absence of any commercial or financial relationships that could be construed as a potential conflict of interest.

## Abbreviations

PTH: parathormone
IOPTH: Intraoperative PTH
CKD: Chronic Kidney Disease
SHPT: Secondary hyperparathyroidism
ΔPTH: Parathormone drop between measures.
